# Regulation of Reactive Oxygen Species and Antioxidant Defense in Plants under Salinity

**DOI:** 10.3390/ijms22179326

**Published:** 2021-08-28

**Authors:** Mirza Hasanuzzaman, Md. Rakib Hossain Raihan, Abdul Awal Chowdhury Masud, Khussboo Rahman, Farzana Nowroz, Mira Rahman, Kamrun Nahar, Masayuki Fujita

**Affiliations:** 1 Department of Agronomy, Sher-e-Bangla Agricultural University, Dhaka 1207, Bangladesh; rakib.raihan1406185@sau.edu.bd (M.R.H.R.); chy.masud3844@sau.edu.bd (A.A.C.M.); khussboorahman1305594@sau.edu.bd (K.R.); farzana.nowroz@sau.edu.bd (F.N.); mirarahman73@gmail.com (M.R.); 2 Department of Agricultural Botany, Sher-e-Bangla Agricultural University, Dhaka 1207, Bangladesh; knahar84@yahoo.com; 3 Laboratory of Plant Stress Responses, Faculty of Agriculture, Kagawa University, Miki-cho 761-0795, Japan

**Keywords:** abiotic stress, antioxidant defense, climate change, hydrogen peroxide, lipid peroxidation, oxidative stress, phytohormones, stress signaling

## Abstract

The generation of oxygen radicals and their derivatives, known as reactive oxygen species, (ROS) is a part of the signaling process in higher plants at lower concentrations, but at higher concentrations, those ROS cause oxidative stress. Salinity-induced osmotic stress and ionic stress trigger the overproduction of ROS and, ultimately, result in oxidative damage to cell organelles and membrane components, and at severe levels, they cause cell and plant death. The antioxidant defense system protects the plant from salt-induced oxidative damage by detoxifying the ROS and also by maintaining the balance of ROS generation under salt stress. Different plant hormones and genes are also associated with the signaling and antioxidant defense system to protect plants when they are exposed to salt stress. Salt-induced ROS overgeneration is one of the major reasons for hampering the morpho-physiological and biochemical activities of plants which can be largely restored through enhancing the antioxidant defense system that detoxifies ROS. In this review, we discuss the salt-induced generation of ROS, oxidative stress and antioxidant defense of plants under salinity.

## 1. Introduction

Abiotic stresses are closely associated with climate change, and they hamper the growth and development of plants; consequently, the yield and quality of crops are also negatively affected. Therefore, the sustainability of global agricultural production is threatened by abiotic stresses [1,2]. Salt stress is one of the detrimental abiotic stresses which greatly reduces crop growth and productivity [3]. Around the world, about 20–50% of irrigated land areas are affected by salt stress [4]. An increase in the salinity level in plant growing media leads to an increase in endogenous sodium (Na^+^) and chloride (Cl^−^) contents. Both Na^+^ and Cl^−^ ions can create life-threatening conditions for plants, but between them, Cl^−^ is more dangerous [5]. Salinity-induced osmotic stress, ionic stress and nutrient imbalance as well as their secondary effects altogether lead to the overgeneration of reactive oxygen species (ROS) [5,6].

Oxygen radicals and their derivatives, called ROS, are produced by different cellular metabolisms in various cellular compartments. The major ROS are hydrogen peroxide (H_2_O_2_), superoxide (O_2_^•−^), singlet oxygen (^1^O_2_) and the hydroxyl radical (^•^OH) [2,7]. Although ROS production is a general phenomenon and a part of cellular metabolism in plants, environmental stresses lead to excess generation of ROS which are not only highly reactive but also toxic in nature and damage lipids, proteins, carbohydrates and DNA [2,8]. Higher concentrations of ROS have an injurious effect on cell organelles and tissues of the shoots and roots [9]. Recent studies revealed that ROS have a dual role in plants [10,11,12]. However, whether they will act as a signaling molecule or as a stressor depends on the crucial equilibrium between the generation of ROS and their scavenging. Due to disruption of the equilibrium between ROS production and antioxidant defense, oxidative stress occurs under abiotic stresses (including salinity). Non-enzymatic as well as enzymatic components of the antioxidant defense system scavenge or detoxify the excessive ROS which mitigates the negative effect of oxidative stress [2,13]. The most investigated major components of the antioxidant defense system are superoxide dismutase (SOD), peroxidase (POD/POX), catalase (CAT), the ascorbate-glutathione (AsA-GSH) cycle enzymes (ascorbate peroxidase, APX; monodehydroascorbate reductase, MDHAR; dehydroascorbate reductase, DHAR; glutathione reductase, GR), peroxiredoxins (PRX), glutathione peroxidases (GPX) and glutathione *S*-transferases (GST), which act in reducing ROS under abiotic stress including salt stress [2,14]. Along with enzymatic components, non-enzymatic components such as GSH, ascorbic acid (Asc), α-tocopherol, flavonoids, carotenoids, alkaloids, phenolic acids and also non-protein amino acids play a vital role in protecting the plant from ROS-induced oxidative stress and in enhancing the tolerance against stress [2]. The amount of ROS at the cellular level determines the destructive or signaling roles of the ROS [2,15]. Moreover, ROS are associated with several processes in plants such as germination and root, shoot, flower and seed development [16].

Most of the recent investigations focused on physiological, biochemical and molecular approaches to enhance salt tolerance in plants by mitigating ROS-induced oxidative stress [17,18,19]. The application of many chemical elicitors and biostimulants improves the plant response to salt stress which results in a reduction in the excessive accumulation of ROS [3,19,20,21,22]. Moreover, overexpression of several transgenes has been proved to enhance the gas exchange activity, photosynthetic activity, biosynthesis of photosynthetic pigments, antioxidant components and abscisic acid (ABA) biosynthesis, stimulate signaling of hormones and also improve ion homeostasis which altogether improve ROS metabolism and salt tolerance [19]. This review focuses on the generation and consequence of ROS production and the role of the antioxidant defense system under salt stress. We also discuss the hormonal and gene regulation associated with ROS metabolism under salinity.

## 2. Types of Reactive Oxygen Species

Oxygen exists in the atmosphere as a free molecule (O_2_), and the ground state of oxygen (triplet oxygen, ^3^O_2_) has two unpaired parallel spin electrons with equal spin numbers which are not reactive in nature [23]. In the aerobic respiration process of plants, the oxygen molecule is the primary acceptor of electrons and is involved in fundamental metabolic and cellular functions such as membrane-linked ATP formation. However, when ^3^O_2_ gains extra energy from metabolic processes (biochemical reactions, electron transport chains, ETC, etc.), it overcomes the spin restriction and converts ^3^O_2_ into ROS [7,23].

**Figure 1:**
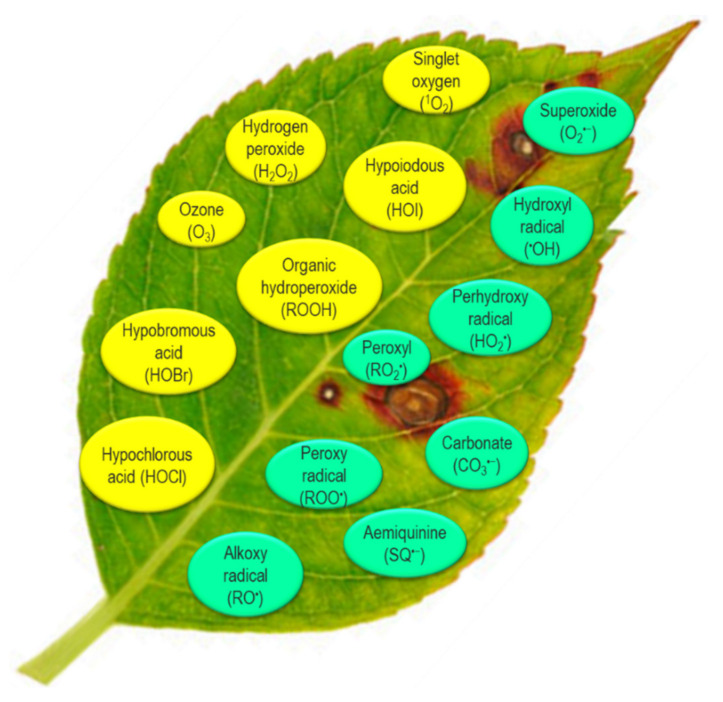
Types of reactive oxygen species (ROS) in plant cells.

**Figure 2:**
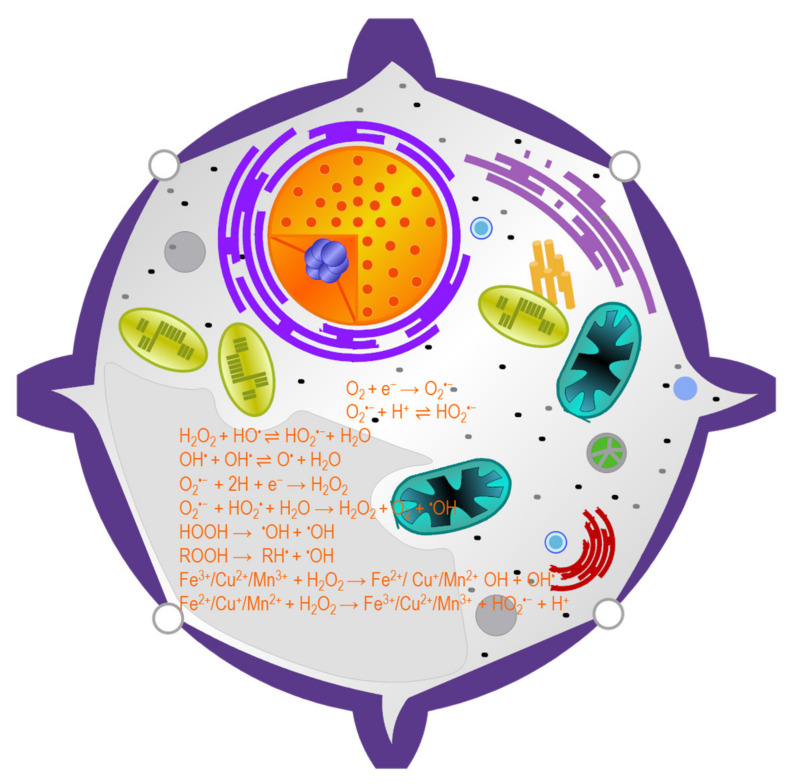
Reactions associated with reactive oxygen species production in plants.

Reactive oxygen species produced in plants are composed of both free radicals and non-radicals [2,13]. The common cellular ROS radicals are O_2_^•−^, ^•^OH, the perhydroxy radical (HO_2_^•^), peroxyl (RO_2_^•^), carbonate (CO_3_^•−^), semiquinone (SQ^•−^), the alkoxy radical (RO^•^) and the peroxy radical (ROO^•^). The non-radical ROS of plant cells are H_2_O_2_, ^1^O_2_, organic hydroperoxide (ROOH), ozone (O_3_), hypoiodous acid (HOI), hypobromous acid (HOBr) and hypochlorous acid (HOCl) (Figure 1; [2,24]).

In plant cells, ROS production occurs due to incomplete or partial reduction of oxygen molecules. Therefore, ROS production is a general phenomenon because they are produced as a result of the oxidation–reduction reaction of several metabolic processes in multiple locations and compartments of plant cells. However, abiotic stress including salinity increases ROS generation which exceeds the equilibrium between antioxidant defense and ROS production [2,7]. In plant cells, a prominent source of ROS production is spilling electrons from the ETC of photosynthesis and respiration, transition metal ion decompartmentalization and redox reactions [7]. Several reactions associated with ROS production are depicted in Figure 2.

## 3. ROS Generation in Plant Cells

**Figure 3:**
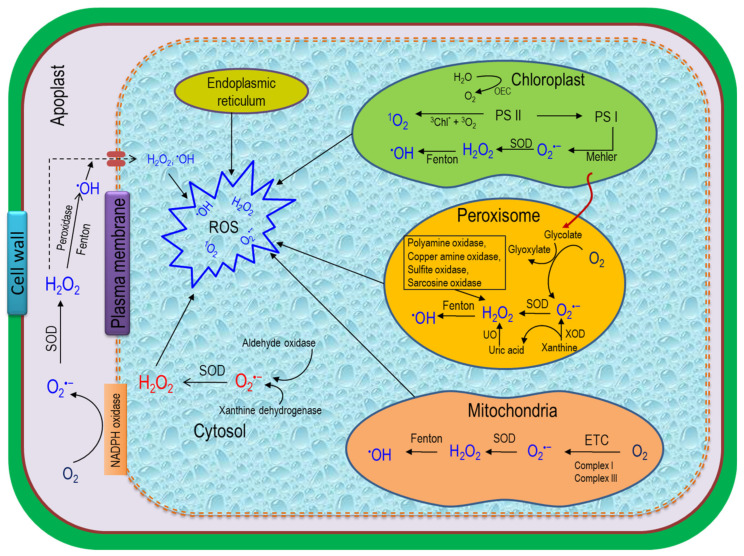
ROS generation process and localization in plant cells. In different cell organelles, ROS are produced through metabolic reactions where different enzymatic and non-enzymatic pathways are involved. ROS—reactive oxygen species; H_2_O_2_—hydrogen peroxide; ^1^O_2_—singlet oxygen; ETC—electron transport chain; ^•^OH—hydroxyl radical; ^3^Chl*—triplet chlorophyll; PS I—photosystem I; PS II—photosystem II; O_2_^•−^—superoxide anion; XOD—xanthine oxidase; SOD—superoxide dismutase; NADPH—nicotinamide adenine dinucleotide phosphate; UO—urate oxidase [24].

Reactive oxygen species are the byproduct of aerobic metabolism in different cell organelles such as chloroplasts, mitochondria, peroxisomes, plasma membranes and cell wall (Figure 3 [24,25]). A specific ROS generation in a cell is highly localized, and different pathways are intensively involved in this process [26,27]. For a better understanding of ROS scavenging tactics in different subcellular compartments, at first, it is obligatory to study the subcellular compartment-specific ROS generation in cells.

Rigid cell wall formation is accelerated under stress conditions when ROS along with POD trigger the polymerization of glycoproteins and phenolic compounds [28]. This cell wall-related POD catalyzes H_2_O_2_ in the presence of NADH, where the NADH is derived from malate dehydrogenase [28]. In addition, diamine oxidases reduce diamines or polyamines (PAs) to quinine and thus produce ROS in the cell wall [29].

Free radicals are also produced in the plasma membrane under abiotic stress. The higher generation of O_2_^•−^ in the plasma membrane is reconciled by NADPH oxidase and quinine reductase where NADPH oxidase transports the electron from the cytoplasmic NADPH and forms O_2_^•−^ which, later on, is converted to H_2_O_2_ [30].

Chloroplasts are considered as the prime site for ROS production that relies on the interactions of chlorophyll (chl) and light [28,31]. Under stress conditions, stomatal conductance (g*_s_*) and the CO_2_ assimilation rate are greatly reduced and thus form excited triplet chlorophyll (^3^Chl*) that impedes in photosynthetic ETC, and promote ROS overgeneration [32,33]. Photosystems I and II (PS I and PS II) are the major sites in chloroplasts where ^1^O_2_ and O_2_^•−^ are largely produced [2]. However, the amount of O_2_^•−^ produced in PS I through the Mehler reaction is converted to H_2_O_2_ with the help of SOD.

In the non-green parts of plants, mitochondria are the major site for ROS generation [2]. ROS produced in mitochondria reduce both mitochondrial energy transportation and other subcellular functions. Respiratory complexes I and III are the main sources of mitochondrial ROS, especially O_2_^•−^. However, the produced O_2_^•−^ in both complexes due to electron leakage is eventually catalyzed by Mn-SOD and Cu-Zn-SOD and produces H_2_O_2_ [33,34].

Another vital site for ROS production is the peroxisomes, where a number of oxidases catalyze different reactions and generate H_2_O_2_ and O_2_^•−^ as byproducts. It is considered that glycolate oxidase (GOX) is the main source of ROS production in peroxisomes [35]. This GOX in peroxisomes causes stomatal closure; as a result, the stomatal gas exchange rate is greatly reduced and thus reduces CO_2_ for RuBisCO generation, causing photorespiration and H_2_O_2_ production [36]. In addition, xanthine oxidase (XOD) activity can also generate O_2_^•−^ and uric acid in the peroxisomal matrix, which are further dismutased to H_2_O_2_ by metalloenzymes SOD and urate oxidase, respectively [37,38].

In the endoplasmic reticulum (ER), both O_2_^•−^ and H_2_O_2_ are produced from the fatty acid oxidation by GOX and urate oxidase activities [39]. In addition, a small amount of O_2_^•−^ is generated in the ER as a byproduct of oxidation and hydroxylation processes which involve cytochrome P450 and cytochrome P540 reductase in the presence of reduced NADPH [27].

Compared to other cell compartments, the ROS production rate is comparatively lower in the cytosol where the redox balance is highly maintained by cytoplasmic NADPH as a central component. However, besides ROS production, the cytosol conducts a pivotal role in the redox signaling process in plant cells. In general, the ROS signaling from different cell organelles passes through the cytoplasm to modulate gene expression in the cell nucleus [40].

Different enzymes contribute to the generation of ROS in apoplasts, among which quinine reductase, NADPH oxidase, SOD and POD are the most important [10,12].

## 4. Consequence of ROS in Plant Cells

**Figure 4:**
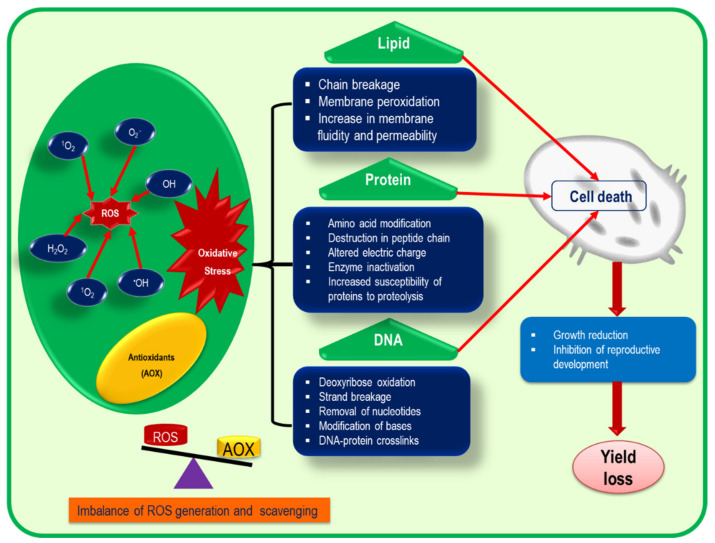
Consequences of oxidative stress on different cellular mechanisms. The imbalance between ROS production and scavenging creates oxidative stress in plants under abiotic stresses. As a consequence, different molecular and cellular damages occur that ultimately cause cell death. ROS—reactive oxygen species; AOX—antioxidants; H_2_O_2_—hydrogen peroxide; ^•^OH—hydroxyl radical; O_2_^•−^—superoxide anion; ^1^O_2_—singlet oxygen.

Overproduction of ROS disrupts the equilibrium between ROS accumulation and scavenging that ultimately damages different cellular biomolecules through protein oxidation, lipid peroxidation, enzyme inactivation, chl degradation and destruction of nucleic acids under stressful conditions (Figure 4; [8,13,41]). However, various factors influence the extent of biomolecular damage which include the concentration of the biomolecule(s), the location of the target biomolecule(s), the site of ROS generation and the reaction rate between the target biomolecule(s) and ROS [13].

The lipid membrane of cells is damaged by oxidative burst, resulting in lipid peroxidation. The extent of peroxidation differs quantitatively between underground and aboveground plant tissues depending on the types of ROS, which is measured by the content of the final product, malondialdehyde (MDA) [29,42]. However, lipid peroxidation not only hampers membrane permeability but also causes electrolyte leakage (EL) and deactivation of enzymes and receptors, as well as accelerating the oxidation process of nucleic acids and proteins [43].

It is well documented that ROS are involved in protein oxidation. However, not all ROS attack all proteins; rather, they cause protein denaturation in a selective manner, among which ^•^OH is the most notorious in nature, causing damage to protein molecules non-selectively [2]. Oxidation of proteins can be both an irreversible and reversible process. Enzymes that contain Fe-S in the center can be damaged irreversibly by the O_2_^•−^ radical, and such type of damage causes cellular dysfunction. On the contrary, glutathionylation and *S*-nitrosylation are reversible changes that can mediate the redox regulation in plants [44].

Nucleic acids are the structural components of proteins and DNA which are rapidly oxidized by the action of ROS. Both chloroplastic and mitochondrial DNA are largely oxidized, compared to that of nucleus DNA, due to their proximity to the ROS production site. Among free radicals, ^•^OH is the most pernicious damaging radical for DNA that modifies nucleotide bases (purine and pyrimidine) by oxidizing the sugar residues in the DNA strands [45]. The DNA replication or transcription process is permanently ceased if the damage induced by ROS is completely irreparable [46]. Consequently, many biochemical processes such as irregular protein synthesis and damage of photosynthetic proteins are directly arrested by DNA damage. In addition, signal transduction, transcription, replication errors and, as a whole, genetic instability are the common fate of DNA due to oxidative stress [13].

In plants, the glycolysis pathway and the enzymes of the TCA cycle are the first targets of a free radical attack. For instance, glyceraldehyde 3-phosphate dehydrogenase and fructose-1,6-bisphosphate aldolase are two common enzymes of the pentose phosphate pathway which are inhibited while ROS production is increased due to oxidative stress [2,47].

## 5. Plant Responses to Salinity

**Figure 5:**
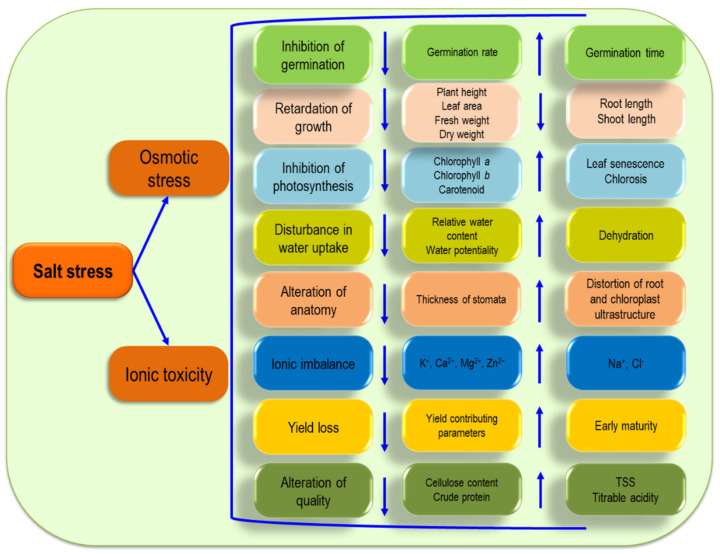
An overview of salt stress-induced changes in plants. TSS—total soluble solids.

Plants are posed with salt stress through two mechanisms. One is osmotic stress, which is a rapid mechanism (within minutes to days), hinders the water uptake and is responsible for stomatal closure, ultimately reducing cell expansion and division. Additionally, the other mechanism, which is slower (days to weeks), is ionic toxicity, which creates an ionic imbalance, disrupts ionic homeostasis and cellular functions and also causes premature senescence. Salt stress is detrimental to plants during the germination, growth and development stages and results in significant yield reduction (Figure 5). Excess accumulation of the salt concentration initiates changes in the mineral distribution, vulnerability of the cell membrane, loss of integrity and a reduced turgor pressure as a result of ionic disequilibrium [48], and, in the extreme case, salt stress results in plant death [14].

Salinity imposes both osmotic stress and ionic toxicity on plants by impairing water uptake, stomatal opening and the ionic balance. As a result, the morphological, physiological and anatomical characteristics of plants start to show changes negatively and finally cause yield loss.

### 5.1. Germination

Salinity reduces the germination of seeds by its osmotic properties and toxicity mechanism at the time of germination. Moreover, seedling establishment is also greatly hampered in salinity as it impedes imbibition, disturbs active metabolism and hinders embryonic tissue development [49]. In *Oryza sativa*, salt stress can cause an adverse effect on the germination stage by inhibiting gibberellic acid (GA) activity in the seed, which, in turn, can curtail seed germination by up to 71% [50]. A similar trend of decline was reported by Shu et al. [51], where it was explained that salt stress favors ABA synthesis while inhibiting the GA synthesis pathway. This phenomenon creates an imbalance in the GA/ABA ratio and in turn, the GA content is decreased, which is beneficial for the seed germination process [52]. During germination, a higher concentration of salt causes osmotic stress in water-deficient conditions that can reduce the *Triticum aestivum* germination rate [53]. Moreover, high salinity has the potential to increase the mean germination time while decreasing the germination rate and, subsequently, lower the germination percentage of *Helianthus annuus* L. [54].

### 5.2. Growth

Salt stress hampers cell prolongation in growing tissue, which, in turn, reduces the leaf area and dry matter assimilation in the plant [5]. In the case of long-term salinity, the plant exhibits a lower photosynthesis rate, lower nutrient storage and less growth hormones [55]. Growth reduction upon salt exposure of seedlings can be found in the reduced root and shoot weights of *Solanum lycopersicum*. Additionally, the damage is more prominent in the root fresh weight (FW) and dry weight (DW) by 40 and 35%, respectively, compared to the shoot FW and DW by 19 and 29%, respectively [56]. Accumulation of Na^+^ and Cl^−^ in leaf tissue modified plant growth hormones, enzyme activity, stomatal closure and photosynthetic activity that resulted in lower assimilation of CO_2_ and, finally, a reduced plant height and DW under salinity [57].

### 5.3. Photosynthesis

Salinity generates an unfavorable condition for plant photosynthesis by affecting g*_s_*, sap flow and the transpiration rate (T*_r_*) through accumulating higher concentrations of NaCl within plant cells [58]. Shortage of the net photosynthesis rate (P*_n_*), T*_r_* and g*_s_* resulted in a reduction in the chl *a*, chl *b* and carotenoid contents in *S. lycopersicum*, and, finally, photosynthesis was hampered to a great extent [59]. A decline in the chl *a*, chl *b* and carotenoid contents has also been recorded in *T*. *aestivum* [60] and *S. lycopersicum* [61] along with a reduced chl *a+b* content. In the salt stress condition, the plant develops an increased chl *a/b* ratio compared to the stress-free condition, and this imbalanced condition triggers reduced photosynthesis in plants [62]. Importantly, salinity can develop a physiological drought condition in the plant, which acts on stomatal closure and lessens the photosynthetic CO_2_ assimilation as well [63]. Plants exhibit chl degradation even at short exposure to salinity and can reach a high severity level with the prolongation of stress [64].

### 5.4. Ionic Imbalance

Ionic imbalance or toxicity is known as a secondary effect of salt stress that causes disturbance in the plant life cycle. Upon salt exposure, imbalance of nutrition is presumed as a primary phenomenon as it can hamper the supply, uptake and translocation of nutrients in plants [65]. Salinity has the capacity to depolarize the root plasma membrane while inducing the guard cell outward-rectifying K channels, which, in turn, results in a higher Na^+^ content with a lower K^+^ content in the plant [66]. Excess Na^+^ increased the Na^+^/K^+^ ratio while decreasing calcium (Ca^2+^) and Mg^2+^ in both the roots and shoots of *S. lycopersicum* seedlings [56]. A similar trend was recorded in *Lens culinaris* [64] and *Vigna radiata* [67]. An excess Na^+^ concentration causes disturbance in plant metabolic activities by replacing the K^+^ content from the enzymatic components of the cell [68]. Moreover, lower uptake of K^+^, Ca^2+^ and Mg^2+^, caused by excess Na^+^ accumulation, subsequently induces an imbalance in mineral homeostasis and ionic stress in the plant [61,69]. Rahman et al. [70] found that under salt stress, ion homeostasis was hampered in *O. sativa* seedlings by the increment in Na^+^ and the Na^+^/K^+^ ratio, compared to the stress-free condition. In this study, the shoots exhibited a higher concentration of Na^+^ than the roots. Along with the reduction in K^+^, Ca^2+^ and Mg^2+^, salt stress also negatively affected the zinc (Zn) content, both in the roots and shoots of the plant.

### 5.5. Water Relation

At a low level of salinity, the plant may not show a difference in water uptake, but at high levels of salt stress, the plant, especially its shoot, is found to be injured greatly with a reduced relative water content (RWC) and a dehydrated condition at the cellular level [71]. *S. lycopersicum* was recorded to be affected by this osmotic stress under salinity, expressed through a reduced RWC, which was also found to be restored after the recovery period [61]. In addition, nutrient imbalance is a common phenomenon in salt stress conditions, caused by reduced water uptake and translocation, as a consequence of accumulated Na^+^ and Cl^−^ ions in the cytoplasm [72]. A reduction in the RWC was also reported in *T. aestivum* [60], *O. sativa* [70,73] and *Sorghum bicolor* [74].

### 5.6. Anatomical Modifications

Besides morphological and physiological changes, salinity may also alter the anatomical characteristics of a plant through modifying the cell wall and nucleus components and leaf structure, especially the ultrastructure of leaf chloroplasts. Due to the close proximity to the saline-affected soil, salt-induced roots were seen to be hampered by the disrupted nuclei and nuclear membrane of *T. aestivum* [75]. Similar results were recorded in *S. lycopersicum* [76], where salinity changed the root structure through decreasing different layers of the columella, cortex cells and cell sizes and enlarging cell nucleoli, ultimately leading to transforming the cell shape and vacuoles. Interestingly, salt-induced *Brassica napus* roots were seen to produce an apoplastic barrier near the root apex to moderate the over-accumulated toxic ions [77]. Salinity causes an alteration in leaf structure by reducing the thickness of the epidermis and mesophyll and, consequently, causes disturbance in water uptake and turgidity [78]. The stomatal frequency was found to be reduced by 10.5 and 22% in *B. juncea* L. [79] and *T. aestivum* [80], respectively, at the salt-induced condition, with a partially closed stomatal aperture, compared to the stress-free condition. Upon salt exposure, the chloroplast ultrastructure was recorded to change by distorting thylakoids and compressing granular and stroma lamellae with massive plastoglobuli, subsequently resulting in a reduced photosynthetic pigment content of the plant [81].

### 5.7. Crop Yield

Salt stress causes alterations in the morphological and physiological characteristics of plants and, subsequently, reduces the yield to a great extent. For example, upon exposure to salt, the yield-contributing characteristics of *H. annuus*, e.g., head diameter, 100-seed weight and oil percentage, were found to be reduced 24, 28 and 5%, respectively, with a severe (26%) seed yield reduction [82]. A similar trend was found in *Hordeum vulgare*, where the tiller number, spike length and 100-grain weight were reduced by 53, 40 and 41%, respectively, in saline conditions [83]. Apart from this, the salt-induced condition interrupts the fertilization process at the time of grain filling through reducing pollen viability and stigma receptivity along with providing minimized photoassimilates. This incident ultimately leads to a yield reduction in the grains [84].

### 5.8. Crop Quality

Salinity causes variation in the qualitative characteristics of plants such as the sugar, citric acid, cellulose and oil contents according to the level of concentration. Upon salt exposure, the cellulose content and sucrose movement are greatly hampered [85]; as cellulose deposition is known as the prime component of fiber quality, this reduction affects the fiber quality in a dose-dependent manner. Salt-induced *Mentha piperita* (a beneficial medicinal and aromatic plant) was seen to lose quality in respect to essential oil and menthol contents compared to the control condition as salinity hampers its growth, photosynthesis and nutrient content to a great extent [86]. Salt-induced *S. lycopersicum* performed positively in respect to electrical conductivity (EC), total soluble solids (TSS), titratable acidity, citric acid content and oxide reduction potential (ORP) [62,87]. As plants produce more soluble sugars and organic acids to compete with the ionic toxicity, generated from Na^+^ and Cl^−^ ions, this can be the reason behind the increment in EC, TSS and titratable acidity in *S. lycopersicum* [88]. Moreover, in saline conditions, plants exhibit early maturation, which promotes sugar accumulation within the fruit and, subsequently, increases TSS [89].

## 6. Salinity-Induced Oxidative Stress in Plants

**Figure 6:**
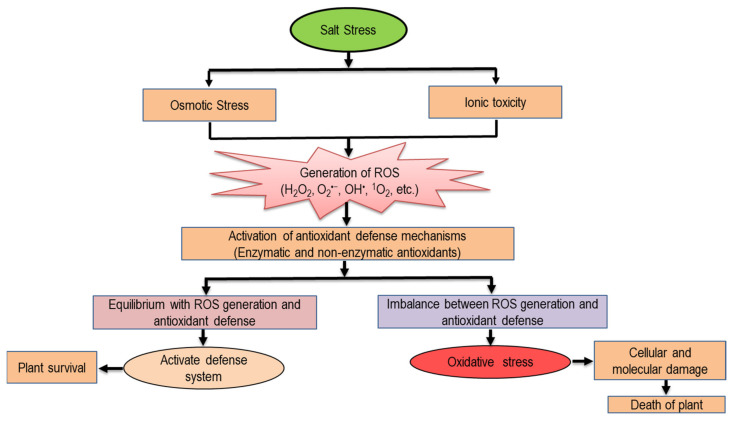
Oxidative stress and antioxidant defense under salinity.

When plants are exposed to salinity, the concentration of Na^+^ and Cl^−^ ions increases abundantly in the soil, which are accumulated at a higher rate, thus reducing the essential ions in plants. Hereby, it disrupts the plant–water relation by creating drought-like conditions, resulting in osmotic stress which is liable for the reduction in g*_s_* and photosynthetic enzyme activities, leading to ROS generation in plants [90]. Besides this, it also triggers ionic stress by inhibiting K^+^ accumulation and interrupting the nutrient balance in plants; thus, by altering redox homeostasis, salinity hampers the flow of electrons from central transport chains to oxygen reduction pathways in different organelles, which leads to the overgeneration of ROS in plants [14]. Other activities such as carbon metabolism, ion redistribution, ABA accumulation and alkalization are also interfered with upon exposure to salinity and foster ROS generation in plants (Figure 6; [91]).

These overproduced ROS are accountable for the damage of nucleic acids and oxidation of different biomolecules, i.e., proteins, lipids, carbohydrates and DNA. Thus, fluctuations occur in their functions and properties, leading to physiological and biochemical alterations and, ultimately, creating oxidative stress in plants, Table 1 [6,10]. Mohsin et al. [60] reported that when *T. aestivum* seedlings are exposed to salt stress at the rate of 150 mM NaCl (mild) and 250 mM NaCl (severe) for 5 d, MDA and H_2_O_2_ contents were increased by 63 and 116%, and 78 and 108%, respectively, compared to the unstressed seedlings. Similarly, Siddiqui et al. [92] showed increased MDA (by 116%), H_2_O_2_ (by 198%) and O_2_^•−^ (by 263%) under salt-stressed (100 mM NaCl) seedlings of *S. lycopersicum*, while increased H_2_O_2_ by 58 and 97% and MDA by 74 and 113% were found under 100 mM and 160 mM NaCl stress, respectively, compared to the controls [56]. Recently, a contrasting response was observed by Ali et al. [93], where the MDA content was decreased but H_2_O_2_ was increased upon exposure to salt stress (150 mM NaCl) in *S. lycopersicum* plants. The extent of damage caused by salinity-induced oxidative stress could be varied among different portions of plants. In most cases, the roots suffer more compared to other tissues as they are directly in contact with the saline conditions, followed by the shoots and leaves [94]. Upon exposure to 100 mM NaCl, a greater increase in the MDA content (by 116%) was observed in the roots than the leaves (by 106%), whereas the H_2_O_2_ content was greater in the leaves (by 149%) than the roots (by 34%), compared to untreated *S. bicolor* plants [95]. Similarly, Jabeen et al. [96] observed a higher amount of MDA content in the roots (44%) than the leaves (38%) compared to the control when *Glycine max* plants were exposed to 100 mM NaCl. Derbali et al. [97] evaluated four genotypes of *Chenopodium quinoa* (cvs. Tumeko, Red Faro, Kcoito and UDEC-5) under different doses of salt (100, 300 and 500 mM NaCl) and reported that both the MDA and H_2_O_2_ contents increased in a dose-dependent manner in these four genotypes. However, the lowest accumulation of MDA and H_2_O_2_ was observed in the salt-resistant genotype (cv. UDEC-5) compared to the salt-sensitive genotypes (cvs. Tumeko, Red Faro, Kcoito) at different NaCl levels. Derbali et al. [98] further reported that the post-stress recovery capacity was higher in the salt-resistant genotype of *C. quinoa* (cv. UDEC-5) compared to the salt-sensitive genotype (cv. Kcoito). From the above-mentioned examples, it can be stated that plant responses against salinity vary among species, and also different genotypes/varieties of the same species.

## 7. Antioxidant Defense System in Plants under Salinity

**Figure 7:**
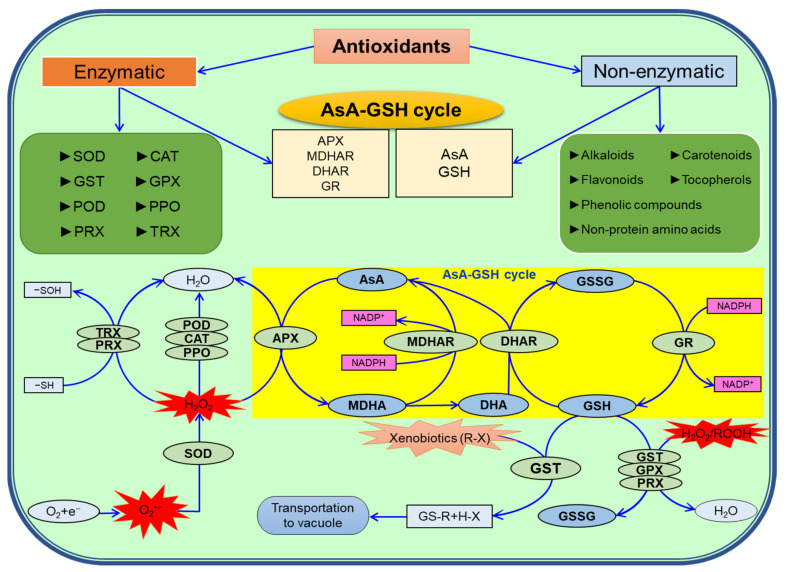
Overview of different types of antioxidants and their combined mechanisms [2]. Detail descriptions are provided in the text. SOD—superoxide dismutase; CAT—catalase; POX—peroxidases; AsA—ascorbate; DHA—dehydroascorbate; GSSG—oxidized glutathione; GSH—reduced glutathione; APX—ascorbate peroxidase; MDHA—monodehydroascorbate; MDHAR—monodehydroascorbate reductase; DHAR—dehydroascorbate reductase; GR—glutathione reductase; GST—glutathione *S*-transferase; GPX—glutathione peroxidase; PPO—polyphenol oxidase; PRX—peroxiredoxins; TRX—thioredoxin; NADPH—nicotinamide adenine dinucleotide phosphate; O_2_—oxygen; e^−^—electrons; H_2_O_2_—hydrogen peroxide; O_2_^•−^—superoxide anion; R—aliphatic, aromatic or heterocyclic group; X—sulfate, nitrite or halide group; ROOH—hydroperoxides; -SH—thiolate; -SOH—sulfenic acid.

Plants have antioxidants that are employed in scavenging ROS by working in coordination with non-enzymatic antioxidants (ascorbate (AsA), GSH, phenolic compounds, flavonoids, alkaloids, α-tocopherol, non-protein amino acids, etc.) and enzymatic antioxidants (SOD, APX, CAT, DHAR, MDHAR, GPX, GR, GST, POD/POX polyphenol oxidase, PPO, PRX, thioredoxin (TRX), etc.), and that protect them from oxidative damage (Figure 7; [13,24]).

In plants, SOD is the major antioxidant that activates the first line of defense against ROS-induced damage by converting O_2_^•−^ into H_2_O_2_; this is further accumulated by CAT, APX, GPX, PPO, PRX and TRX enzymes or catalyzed in the Asada−Halliwell pathway (AsA-GSH cycle) [124,125]. To detoxify ROS in the AsA-GSH cycle, plants have non-enzymatic antioxidants (AsA and GSH) and four enzymatic antioxidants (APX, DHAR, MDHAR and GR); thus, by minimizing ROS, they can maintain redox homeostasis in plants, Table 2 [2,8,24]. Furthermore, H_2_O_2_ and other xenobiotics are also detoxified with the help of the GST and GPX enzymes [126,127]. Many researchers have proposed that the activities of these antioxidants depend on the salinity threshold, duration of salinity exposure and growth stages of plants [128]. For instance, Ali et al. [129] found altered antioxidant enzyme activities in scavenging ROS under two concentrations of NaCl (80 mM and 160 mM), with a maximum reduction in SOD, POD, APX and CAT of 49, 43, 39 and 52% in cv. P1574, and 67, 46, 47 and 61% in cv. Hycorn-11, respectively, which are two *Z. mays* cultivars, at 160 mM NaCl stress and concluded that the P1574 cultivar is more salt-tolerant than the Hycorn-11 cultivar. Similarly, upon exposure to 100 mM NaCl, total phenols (by 60%) and AsA (by 55%) were increased in *S. lycopersicum* plants together with enhanced SOD, CAT, POD and PPO activities [130]. Jiang et al. [131] highlighted the *TaSOS1* gene in response to salt stress in two spring *T. aestivum* genotypes, Seri M82 (salt-sensitive) and CIGM90.863 (salt-tolerant), and observed a higher expression of most of the 18 *TaSOS1* genes in the roots of salt-tolerant seedlings than the salt-sensitive seedlings. Recently, several exogenous protectants (i.e., GA, salicylic acid (SA), melatonin (MT), silicon, selenium) have been used to upregulate the antioxidant machinery in plants under salinity [132,133,134,135]. For instance, Ahmad et al. [133] found increased activities of SOD (9%), APX (13%), CAT (26%), GR (40%) and POD (98%) when seed priming was conducted with 0.5 mM GA. Foliar spraying of SA (0.5 mM) enhanced AsA, GSH, total phenols and anthocyanin biosynthesis and increased SOD and CAT activities, thus reducing the H_2_O_2_ and O_2_^•−^ content in salt-stressed seedlings of *Vicia faba* [134]. Combined application of silicon and selenium has been reported to increase CAT, GR, SOD and APX activities and synthesis of AsA and GSH [135]. Pretreatment of seeds of *Avena nuda* with MT (50 or 100 µM) upregulates CAT, APX, POD and SOD activities, thus reducing H_2_O_2_ (by 34%), O_2_^•−^ (by 26%) and MDA (by 51%), compared to salt-treated plants [132]. Chen et al. [136] also reported that seed priming with MT reduces the accumulation of H2O2 and MDA and the percentage of EL and enhances the germination rate under salt stress conditions. Furthermore, Zhang et al. [137] observed that both foliar and root application of MT (100 µM) increased SOD, POD, CAT and APX activities under salt stress and decreased H2O2, O2•− and MDA accumulation in Beta vulgaris.

## 8. Signaling of ROS in the Regulation of Salinity

**Figure 8:**
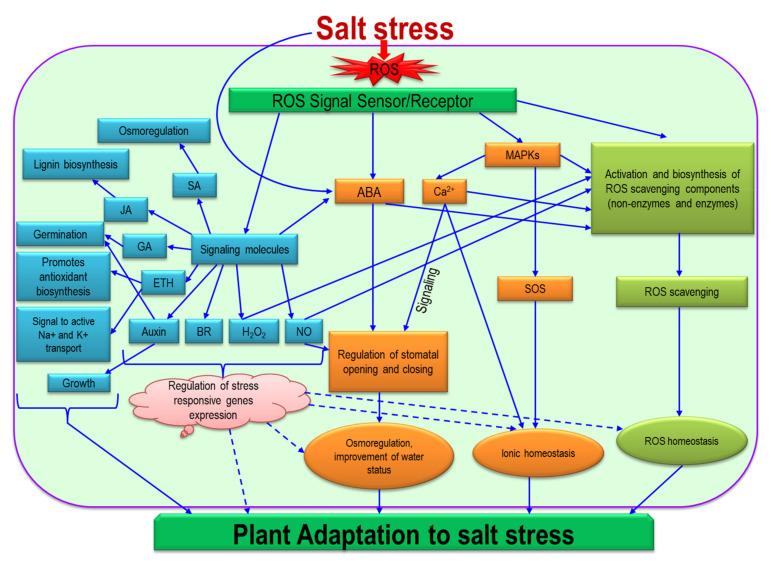
Common proposed ROS signaling pathways during plants’ response to salt stress. Under salt stress, ROS production induces mitogen-activated protein kinase (MAP) cascades which mediate different adaptive responses. MAPs, nitric oxide (NO), Ca^2+^ and other signaling molecules have been suggested to be connected in activating antioxidant defense, stomatal movement, membrane properties and ionic homeostasis. The salt overly sensitive signaling (SOS) pathway interacting with other pathways functions in maintaining ionic homeostasis. The ROS-induced ABA production and ROS-induced activation of MAPs and Ca^2+^ signal regulate stomatal opening and closing. ROS signal sensors/receptors can induce activation of biosynthesis/functioning of different hormones and signaling molecules such as salicylic acid (SA), jasmonic acid (JA), gibberellic acid (GA), ethylene (ETH), auxin, brassinosteroid (BR), H_2_O_2_ and NO. These hormones/signaling molecules can interact with ROS, single or multiple hormones or signaling molecules; can interact with various signal cascades/pathways; and can regulate stress-responsive gene expression which modulates various metabolic and physiological functions, contributing to plant adaptation to salt stress. Dashed lines indicate the mechanisms which are not yet identified.

Salt stress directly induces primary stresses such as osmotic and ionic stresses as well as imposing secondary stresses such as oxidative stress caused by ROS. These ROS play a signaling function at a certain low concentration which is variable depending upon many factors [139]. In recent decades, research on salt stress signals and adaptation proposed different pathways which still remain hazy because of the complex interaction between and among biomolecules (Figure 8; [139,140,141]). Under salt stress, ROS production induces mitogen-activated protein kinase (MAP) cascades which mediates ionic, osmotic and ROS homeostasis [139,142].

The salt overly sensitive signaling (SOS) pathway functions in maintaining ionic homeostasis, through extruding Na^+^ into the apoplast. Under salinity, excess intracellular or extracellular Na^+^ triggers, and ROS-induced MAPs stimulate, a Ca^2+^ signal and production. Ca^2+^ together with the SOS pathway excludes Na^+^ to maintain ionic homeostasis [141,143,144]. ROS signals stimulate antioxidant defense and ROS scavenging which confer biomembrane protection and restore membrane function for maintaining ionic and nutrient homeostasis [141,144].

The ROS-induced ABA production and ROS-induced activation of MAPs and the Ca^2+^ signal regulate stomatal opening and closing. The osmoregulation and plant water status are maintained properly as a result [140,142]. Nitric oxide is also considered as one of the regulators of stomatal opening and closing [145]. ROS signal sensors/receptors can induce SA which also has a role in osmoregulation. Different signaling molecules or hormones can be stimulated by ROS which have diversified physiological, growth and developmental functions in the salt adaptation of plants. The ROS signal induces JA to modulate lignin biosynthesis, and GA to affect germination, and it induces auxin to modulate growth [146]. Ethylene–ROS interaction has a differential function. Ethylene enhances ROS generation that activates Na^+^ and K^+^ transport, where expression of different genes is involved. The modulation of ETH signaling controls AsA biosynthesis under salinity. The synchronizing action of ABA and ETH modulates the AsA biosynthesis during salt exposure. The Ca^2+^ signaling persuaded by ROS signaling participates in AsA biosynthesis [3]. Several research reports demonstrated that exogenous application of hormones and signaling molecules regulated or enhanced the antioxidant defense system, conferred osmoprotection and improved physiology, which defended against oxidative damage under salt stress conditions [147,148,149]. There might be other hormones/hormone-like molecules or signaling molecules in this complex and hazy salt stress adaptation pathway. Knowledge on the signaling functions of ROS is misty as it is connected to the signaling function and biological function of different biomolecules. The dual role of ROS—creating oxidative stress and having a signaling function—is well known, but the mechanisms, interplay and cross-talk with other molecules and components are complex and yet to be discovered.

## 9. Hormonal Regulation and ROS under Salinity

**Figure 9:**
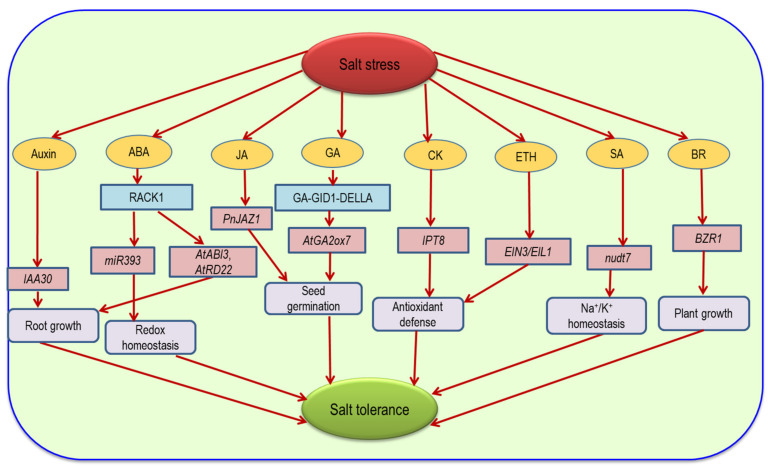
Overview of major plant hormone regulation in *Arabidopsis* under salt stress where different salinity-mitigating traits of phytohormone-regulated genes help to induce salt tolerance in *Arabidopsis*. Detailed descriptions are provided in the text. ABA—abscisic acid; JA—jasmonic acid; GA—gibberellic acid; CK—cytokinin; SA—salicylic acid; ETH—ethylene; BR—brassinosteroid; RACK1—receptor for activated C kinase 1.

Plant hormones or phytohormones are chemicals that are present in plants in small amounts but have a great impact on plant growth and development. Plant hormones play different biological roles in the presence of environmental stress and regulate plant growth positively or negatively. Under salinity, diverse signaling pathways coordinately work to mitigate salt stress [150]. Flexible regulation of phytohormone signaling helps plants to adapt under salinity (Figure 9).

In plants, ABA is an important stress-responsive hormone that plays a crucial role in osmotic stress and especially in ROS-mediated signaling pathways. Salt-induced osmotic stress in plants causes a higher accumulation of ABA [151,152]. This higher accumulation of ABA helps to mitigate the negative effect of salinity by improving photosynthesis and the osmolyte content and reducing ROS-mediated toxicity [153]. It also triggers the accumulation of K^+^ and Ca^2+^ which inhibit the uptake of Na^+^ and Cl^−^ [154]. ABA also regulates Na^+^/K^+^ homeostasis and the H_2_O_2_ content under salt stress [155]. Abscisic acid is known as a vital salinity-mediated signal which regulates salinity-responsive genes. Overexpression of the *MAX2* gene increased the resistance to salt stress, which was associated with redox homeostasis and ABA [156]. Osmotic homeostasis is regulated by ABA-activated SnRK2s which are responsible for the breakdown of starch into sugar and derivate osmolytes [157]. To scavenge ROS, ABA-generated H_2_O_2_ triggered the accumulation of NO and activated protein kinase (MAPK) [139]. The MAPK cascade signaling upregulates the antioxidant genes and acts against salinity [158]. Salt stress reduced the lateral root length in plants which mediated ABA signaling [159]. Similar to ABA, auxin also alters the root apical meristem under salt stress. In *Arabidopsis thaliana*, receptor for activated C kinase 1 (RACK1) is involved in the biosynthesis of the *miR393* gene which mediates both ABA and auxin regulation to induce redox homeostasis and alter lateral root growth under salinity [160]. During salt exposure, accumulation of excess ROS altered the auxin distribution, induced oxidative damage and caused a reduction in the root apical meristem [161]. Seed germination modulated membrane-bound transcription factor (NTM2), altered auxin signaling in plants which caused overexpression of the *IAA30* gene and induced salt tolerance in the *Arabidopsis* plant [162]. Plant growth and development depend on seed germination which is closely related to GA. DELLA proteins act as a growth suppressor of plants under salt stress. GA binds with the GIBBERELLIN INSENSITIVE DWARF1 (GID1) receptor which recruits DELLA proteins and forms the GA-GID1-DELLA complex [163]. Thus, GA reduces DELLAs and salt tolerance in plants. Some GA-regulated genes, viz., *AtGA2ox7* and *OsGA2ox5*, also help to increase salinity tolerance in *A. thaliana* and *O. sativa* plants, respectively [164,165].

Under the saline condition, plants regulate CK production along with ROS homeostasis to induce salt tolerance in plants. CK is a master phytohormone which acts as a free ROS scavenger and opposite to ABA and induces salt tolerance in plants [166]. CK receptor genes, viz., *AHK2*, *AHK3* and *AHK4*, help to modulate the stress signal in plants which regulates the osmolyte content and membrane integrity [167]. Histidine kinase (HK) of CK receptors plays an important role in CK regulation and stress responses [168]. In the presence of CK, receptor gene *CRE1* changes to its HK form and acts as a negative regulator of stress signaling. However, in the absence of CK, it converts into its phosphatase form and does not show any negative reactions [169]. Due to the higher CK content, receptor genes *AHK2* and *AHK3* antagonistically regulate ABA synthesis in *Arabidopsis* and consequently inhibit the ABA action in germinating seeds and seedling growth through the CK receptor HKs [170]. However, ROS production is reduced by the overexpression of CK biosynthetic gene *IPT8* which helps to upregulate antioxidant enzyme activity under salinity in *Arabidopsis* [171]. Likewise, JA also works against salt stress and helps to mediate ROS. A high concentration of JA was noticed in salt-treated plants, which helps to reduce oxidative damage and shows a positive relationship with salt tolerance [172]. Qiu et al. [173] also observed an increase in the salt tolerance of *T. aestivum* by exogenous application of JA through the reduction in the Na^+^ content, ROS and lipid peroxidation. The salt tolerance mechanism of JA is modulated by jasmonate ZIM domain (JAZ) transcription factors which repress MYC2 transcription factors. These MYC2 transcription factors are used by both JA and ABA to regulate salt responses where the functioning of JA activates ABA-regulated genes *RD22* and *AtADH1* under salt stress to induce salt tolerance [173]. In *Arabidopsis*, the *PnJAZ1* gene regulates the JA pathway to induce salt tolerance and cause upregulation of ABA-regulated genes, viz., *AtABI3* and *AtRD22*, to regulate seed germination and seedling growth under salinity [174].

In plants, salinity tolerance is induced by SA via activating the GORK channel which helps to maintain the membrane potential and K^+^ loss in plant cells [175]. It also upregulates the activity of the H^+^-ATPase enzyme which helps in K^+^ retention in the saline condition. However, SA did not retard the accumulation of Na^+^ in plant roots but, instead, helped to reduce the Na^+^ concentration in the roots [176]. In *Arabidopsis*, expression of the *nudt7* gene reduced the shoot Na^+^ concentration during prolonged salt stress [177]. In barley, the application of SA helps to increase P*_n_*, the carotenoid content and membrane integrity and to reduce ROS toxicity which ultimately helps to increase the K^+^ and osmolyte concentration in plant roots [178]. It also caused overexpression of the *P5CS* gene which causes Pro accumulation and thus induces salt tolerance in *Dianthus superbus* [179]. ETH homeostasis and signaling have a direct relation with salt stress. Salt stress regulates key components of ETH signaling and also causes overexpression of the *EIN3/EIL1* gene which is known as a defense-related gene. This gene helps to reduce the H_2_O_2_ content and upregulate antioxidant enzymes to induce salt tolerance in *Arabidopsis* [180]. Similarly, brassinosteroid (BR) also helps to mediate salt stress in *S. lycopersicum* by the overexpression of *BRI1* or *BSK5* [181]. In saline conditions, BRASSINOSTEROID INSENSITIVE1 KINASE INHIBITOR1 (BKI1) translocated BRASSINAZOLE RESISTANT 1 (BZR1) and BRI1-EMSSUPPRESSOR 1 (BES1) and caused overexpression of the *BZR1* gene in the *Arabidopsis* plant which controlled plant growth and development [182]. In *Arabidopsis*, BR-mediated salt tolerance signaling is linked with ABA, ETH and SA pathways which indicates the cross-talk of phytohormones in mitigating salt stress [183].

## 10. Gene Regulation for Antioxidant Defense under Salinity

Antioxidant enzymes are considered the first line of defense to eliminate accumulated ROS. Antioxidant enzymes are encoded by different gene families which are located in different parts of the cell. The activities of these enzymes are controlled by the expression of genes encoding antioxidant enzymes which are varied in crops. Mishra et al. [184] observed increased SOD activity in a tolerant cultivar of *O. sativa* which occurred due to the expressions of the *Cu/Zn-SOD* genes. In *O. sativa* (cv. BRS AG), expression of *OsCATB* increased with the duration (10, 15 and 20 d) of salt stress, which resulted in increased activity of CAT during the exposure to salinity [185]. *GPX* genes of *T. aestivum* demonstrated different expression patterns at a stressed condition, where *TaGPX* genes showed higher expression at the leaf developmental stage [186]. Glutathione peroxidase is considered as an important antioxidant enzyme involved in the reduction of H_2_O_2_ with the help of GSH [2]. Salt-tolerant *H. vulgare* genotypes showed higher GPX activity which was regulated by the higher expression of the *GPX* gene [187]. In *B. juncea*, higher expression of ROS detoxification genes, viz., MDHAR and DHAR, was observed in salt-tolerant genotypes compared to the sensitive ones [188]. MDHAR and DHAR help to detoxify plant cells with the help of the AsA-GSH cycle [2]. Under salt stress, antioxidant genes of stressed plants are upregulated or downregulated with the severity of stress. Filiz et al. [189] worked with 36 isoform genes of 10 natural *Arabidopsis* ecotypes under salt stress, where 64% upregulation was demonstrated by *CAT* genes, and 55% downregulation was recorded in *SOD* and *GPX* genes.

Overexpressed genes encoding antioxidant enzymes help to mitigate ROS and induce stress tolerance in plants; some examples are cited in Table 3. Overexpression of *Cu/ZnSOD* in tobacco plants reduced ROS-induced damage in plants and improved the chl content under salinity [190]. Overexpression of *PutAPX* in *A. thaliana* reduced lipid peroxidation and improved plant growth under salt stress [191]. In *PaSOD-* and *RaAPX*-overexpressed potato plants, Shafi et al. [192] observed an improved RWC and chl content, and a reduction in the MDA content, under 150 mM NaCl-induced salt stress.

## 11. Conclusions

The generation of ROS in plants is a natural phenomenon that occurs as a part of different metabolic processes in multiple cellular and subcellular compartments. Salinity-induced osmotic stress and ionic toxicity disrupt redox homeostasis and trigger the overproduction of ROS and ROS-induced oxidative stress under salt stress. Several studies revealed that ROS have both negative and positive roles in plants. Along with signaling, ROS aid several basic processes and pathways in plant cells. Lower concentrations of ROS were found to be essential for proliferative pathway activation, cell differentiation and stem cell renewal. On the contrary, excessive accumulation of ROS at the cellular level acts as a stressor and causes oxidative damage of lipids, proteins, carbohydrates and RNA and DNA. 

As ROS have a dual role in plants, the metabolism of ROS is crucial for growth, development and adaptation under salinity. The antioxidant defense system plays a vital role in ROS metabolism by scavenging and detoxifying ROS. Plant hormones, amino acids and their derivatives, PAs and vitamin supplementation contributed to ROS metabolism and decreased salt-induced oxidative stress under different levels of salinity by upregulating the antioxidant defense system, biological processes, ion homeostasis, osmolytes and gene expression in several studies. In previous investigations, under different levels of salinity, overexpression of genes helped to mitigate ROS-induced oxidative stress. In recent studies, molecular and genetic tools developed transgenic plants with enhanced activities of antioxidant enzymes and ROS detoxification under salinity.

Although the dual role of ROS is known, the metabolism process of ROS under salinity is complex, including the mechanisms, cross-talk with other molecules and components that are yet to be understood. Therefore, further research is required to understand the metabolism process of ROS more clearly. The understanding of ROS metabolism under salinity will be helpful in mitigating salt-induced oxidative stress. The mechanisms, interplay, cross-talk with other molecules and components are complex and warrant further research.

The present review focuses on ROS production and the antioxidant defense system under salt stress where information about cultivated and common plant species was included mostly from recently published research articles. There are salt-tolerant plant species in nature. Halophytes bear unique morphological, anatomical and physiological behavior, and that is why they are adapted to grow in coastal saline soils, mangrove forests and salt-affected lands of arid and semi-arid regions. Understanding the mechanisms of halophytes, finding out the signaling cascades and identifying salt stress-responsive genes can be useful for managing salt stress-affected cultivated and commonly grown plant species through different agronomic, breeding, biotechnological and other approaches.

## Figures and Tables

**Table 1:** Oxidative stress in plants under salinity.

Plant Species	Level(s) of Salt Stress	Oxidative Damage	References
*T. aestivum* cv. Pradip	150 and 300 mM NaCl; 4 d	Lipid peroxidation increased by 60%. H_2_O_2_ level increased by 73%.	[99]
*B. napus* cv. BINA Sharisha 3	100 and 200 mM NaCl; 2 d	MDA increased by 69 and 129%. H_2_O_2_ incremented by 76 and 90%.	[100]
*O. sativa* cvs. MI-48, IR-28 (salt-sensitive)	100 mM saline solution (mixture of NaCl, MgCl_2_, MgSO_4_ and CaCl_2_ salts); 35 d	H_2_O_2_ and O_2_^•−^ generation increased by 2-fold.	[101]
*S. lycopersicum* cv. Chibli F1	120 mM NaCl; 8 d	Lipid peroxidation elevated by 35 (leaves) and 37% (roots).	[102]
*O. sativa* cv. KDML105	60, 120 and 160 mM NaCl; 3 d	H_2_O_2_ increased in a dose-dependent manner.	[103]
*G. max* cv. PK9305	100 mM NaCl; 7 d	Increment in MDA found in both leaves and roots. Higher activity of LOX in roots.	[104]
*Phaseolus vulgaris* cv. Nebraska	2.5 and 5.0 dS m^−1^ (NaCl/CaCl_2_/MgSO_4_ = 2:2:1); 40 d	MDA and H_2_O_2_ increased with increasing saline concentrations. EL increased, but reduced membrane stability index (MSI).	[105]
*B. juncea* cv. Pusa Jai Kisan	100 mM NaCl; 30 d	Higher accumulation of H_2_O_2_ and thiobarbituric acid reactive species (TBARS).	[106]
*S. tuberosum* cv. Hui 2	50, 75 and 100 mM NaCl; 31 d	Elevation in MDA by 164%.	[107]
*O. sativa* cv. BRRI dhan47	200 mM NaCl; 3 d	H_2_O_2_ increased by 82%. Higher production of MDA. The activity of LOX increased by 78%.	[70]
*P. vulgaris* cvs. Tema (high-yielding) and Djadida (low-yielding)	50, 100 and 200 mM NaCl; 7 d	Increase in MDA by 44 (cv. Tema) and 56% (cv. Djadida).	[108]
*O. sativa* cv. BRRI dhan47	150 mM NaCl; 3, 6 d	MDA increased by 80 (3 d) and 203% (6 d). Increment in H_2_O_2_ found, by 74 (3 d) and 92% (6 d). Lipoxygenase (LOX) activity increased by 69 (3 d) and 95% (6 d).	[69]
*V. radiata* cv. BARI Mung-2	200 mM NaCl; 2 d	Upregulation of MDA and H_2_O_2_. The activity of LOX also increased.	[67]
*Pisum sativum* cv. Shubhra IM-9101	100 and 400 mM NaCl; 7 d	Increment in O_2_^•−^ by 171–407%. H_2_O_2_ increased by 191–249%.	[109]
*T. aestivum* cvs. Kharchia local (salt-tolerant) and HD2329 (salt-sensitive)	10 dS m^−1^ NaCl; 7 d	EL increased more in cv. HD2329 (by 2.6- and 1.5-fold in the roots and shoots) than Kharchia local (by 1.9- and 1.4-fold in the roots and shoots). Higher accumulation of H_2_O_2_ and MDA in sensitive cultivar.	[110]
*B. juncea* cvs. CS-52 (salt-tolerant) and RH-8113 (salt-sensitive)	50, 100 and 150 mM NaCl; 21 d	MDA generated more in RH-8113 (138%) than CS-52 (126%). H_2_O_2_ increased in dose-dependent manner in sensitive cultivar, but at 150 mM NaCl, it increased by 33% in CS-52.	[111]
*Cicer arietinum* cvs. Flip 97-43c (tolerant), ICC 4958 (tolerant), Flip 97-97c (susceptible) and Flip 97-196c (susceptible)	100 mM NaCl; 3, 7 and 12 d	Accumulation of MDA began to increase after 12 d by 224% in T1 genotype. In S2 genotype, 2.21-, 8.20- and 10.15-fold upregulation of MDA content was found at 3, 7 and 12 d, respectively.	[112]
*S. lycopersicum* cv. Hezuo 903	150 mM NaCl; 10 d	Elevation in lipid peroxidation by 2.6-fold. H_2_O_2_ increased by 2.5-fold.	[113]
*B. napus* cv. BINA Sharisha 3	100 and 200 mM NaCl; 2 d	Gradual increase in MDA by 60–129% in a dose-dependent manner. Production of H_2_O_2_ elevated by 63–98%.	[114]
*S. lycopersicum* cv. Pusa Ruby	150 mM NaCl; 5 d	Elevation in H_2_O_2_ and MDA. Increased LOX activity.	[115]
*Zea mays* cvs. BARI hybrid Maize-7 and BARI hybrid Maize-9	150 mM NaCl; 15 d	O_2_^•−^ and H_2_O_2_ increased by 130 and 99%. Higher production of MDA by 109%. LOX activity enhanced by 133%.	[116]
*Luffa acutangula* cv. Pusa Sneha	100 mM NaCl, 10 d	Increase in H_2_O_2_, O_2_^•−^, EL, MDA and LOX activity by 140, 145, 251, 358 and 157%, respectively.	[117]
*Gossypium hirsutum*	150 mM NaCl; 3, 6, 9 and 12 d	Increased H_2_O_2_ by 58% (3 d), 34% (6 d), 45% (9 d) and 37% (12 d). O_2_^•−^ incremented by 47% (3 d), 25% (6 d), 37% (9 d) and 32% (12 d). MDA augmented by 25% (3 d), 27% (6 d), 36% (9 d) and 41% (12 d).	[118]
*T. aestivum*	100 mM NaCl; 1 d	1.5- and 1.2-fold upregulation of EL and MDA.	[119]
*T. aestivum* cv. Norin 61	250 mM NaCl; 5 d	Increase in MDA and H_2_O_2_ by 62 and 35%. EL increased by 130%.	[120]
*G. max* cv. Giza 111	75 and 150 mM NaCl; 56 d	Level of MDA increased by 47 and 75%. H_2_O_2_ augmented by 42 and 50%.	[121]
*Lactuca sativa* cv. SUSANA	4 dS m^−1^ (low) and 8 dS m^−1^ (high) NaCl; 60 d	MDA increased by 44% under low level of NaCl, while increased under high NaCl (70 and 87%) in both seasons. H_2_O_2_ augmented by 183 (high) and 166% (low) in both seasons. O_2_^•−^ increases more at high dose (208 and 262%) in both seasons.	[122]
*L. culinaris* cv. BARI Masur-7	110 mM NaCl; 2 d	Content of MDA and H_2_O_2_ increased by 164 and 229%.	[123]

**Table 2:** Activities of antioxidant defense system against salinity.

Plant Species	Level(s) of Salt Stress	Antioxidant Defense	References
*G. max* cv. A3935	50 (low), 100 (medium) and 150 (high) mM NaCl; 30 d	High salinity reduces SOD activity in roots (28%) and leaves (38%). APX activity decreased in leaves by 20% (low) and 57% (high), but slightly increased in roots at low salinity (10%) and decreased by 29% under high salinity. GR and CAT activities decreased in both roots and leaves under high salinity.	[138]
*T. aestivum* cv. Pradip	150 and 300 mM NaCl; 4 d	AsA content sharply decreased. GSH content and GSH/GSSG ratio increased. Slight increase in APX and GST activities, whereas activities of CAT, DHAR, MDHAR, GR and GPX decreased at 300 mM NaCl stress.	[99]
*B. napus* cv. BINA Sharisha 3	100 and 200 mM NaCl; 2d	AsA and GSH content decreased, but GSSG content increased at 200 mM NaCl. GPX, GST and GR activities increased at 100 mM NaCl. Increase in APX activity, and decrease in CAT, DHAR, MDHAR, GR, GST and GPX activities at 200 mM NaCl stress.	[100]
*O. sativa* cv. Pokkali (tolerant)	100 mM saline solution (mixture of NaCl, MgCl_2_, MgSO_4_ and CaCl_2_ salts); 35 d	Increased activities of SOD, CAT, APX and POX.	[101]
*O. sativa* cv. KDML105	60, 120 and 160 mM NaCl; 3 d	SOD, APX and GR activities increase with increasing NaCl concentrations. CAT activity reduced by 1.6-fold at 160 mM NaCl.	[103]
*G. max* cv. PK9305	100 mM NaCl; 7 d	Activities of CAT, SOD and PPO observed more in leaves than roots. Higher activity of POX in roots than shoots.	[104]
*S. tuberosum* cv. Hui 2	50, 75 and 100 mM NaCl; 31 d	SOD activity increased in a dose-dependent manner. CAT and POD activities decreased at 100 mM NaCl stress, but still higher than non-stressed plants.	[107]
*O. sativa* cv. BRRI dhan47	200 mM NaCl; 3 d	AsA content reduced by 49%. Reduction in GSH/GSSG ratio by 42%. The activity of SOD increased by 20%. Reduced CAT activity by 33%.	[70]
*P. vulgaris* cvs. Tema (high-yielding) and Djadida (low-yielding)	50, 100 and 200 mM NaCl; 7 d	GR activity increased by 60% (Tema) and 20% (Djadida). CAT activity increased by 4- and 2-fold in Tema and Djedida. APX activity increased by 9- and 6- fold in Tema and Djedida. Increment in AsA and total flavonoid content in cv. Tema (by 33 and 47%) and cv. Djadida (by 26 and 70%). Total phenolic compounds decreased markedly in cv. Djadida.	[108]
*O. sativa* cv. BRRI dhan47	150 mM NaCl; 3, 6 d	AsA content decreased, but DHA content increased. Higher level of GSH and GSSG content. Upregulation of MDHAR, DHAR and GR activities. APX activity enhanced at 6 d. SOD and GPX activities increased with increasing duration of stress. Reduction in phenolic and flavonoid contents.	[69]
*V. radiata* cv. BARI Mung-2	200 mM NaCl; 2 d	Activities of SOD and GST increased by 49 and 88%. CAT activity reduced by 50%. No significant change was observed in GR and GPX activities. Reduction in MDHAR and DHAR activities.	[67]
*P. sativum* cv. Shubhra IM-9101	100 and 400 mM NaCl; 7 d	Reduction in CAT (94%), POD (57%) and APX (86%) activities. Increase in SOD activity by 174%.	[109]
*B. napus* cv. BINA Sharisha 3	100 (mild) and 200 (severe) mM NaCl; 2 d	AsA content reduced by 44% under severe stress. GSSG content upgraded by 116% under severe stress. APX activity increased, but reduction in MDHAR, DHAR and GR activities. GPX activity reduced under severe stress. The activity of GST enhanced. CAT activity dropped by 32% (mild) and 41% (severe).	[114]
*S. lycopersicum* cv. Pusa Ruby	150 and 250 mM NaCl; 4 d	Upregulation of SOD activity by 30% (150 mM) and 43% (250 mM). CAT and GR activities decreased. Activities of APX, MDHAR, DHAR, GPX and GST upgraded. AsA content reduced, but DHA content increased. Both GSH and GSSG content upgraded.	[61]
*G. hirsutum*	150 mM NaCl; 3, 6, 9 and 12 d	SOD activity enhanced by 47, 37, 26 and 18% at 3, 6, 9 and 12 d, respectively. Higher activity of POD found, by 103, 63 and 11% at different durations. CAT activity increased by 28, 20, 14 and 16%. Activity of APX enhanced by 126, 104, 67 and 37% at 3, 6, 9 and 12 d, respectively.	[118]
*S. lycopersicum* cv. Pusa Ruby	150 mM NaCl; 5 d	AsA content decreased, but DHA increased with ratio lowered by 50%. GSH content reduced, but GSSG content increased with decrease in GSH/GSSG ratio by 45%. Activities of APX, MDHAR, GR, GST and SOD increased by 134, 53, 114, 70 and 16%, respectively.	[56]
*T. aestivum* cv. Norin 61	250 mM NaCl; 5 d	Both AsA and AsA/DHA ratio reduced, but DHA content increased. GSSG content increased, but GSH and GSH/GSSG ratio decreased. Activities of APX, DHAR and GPX increased by 29, 38 and 13%, respectively. Reduction in MDHAR (32%), GR (24%), CAT (37%) and GST (15%) activities.	[120]
*G. max* cv. Giza 111	75 and 150 mM NaCl; 56 d	Total phenol content notably increased by 24 and 33%. AsA content also enhanced by 32 and 64%.	[121]
*L. sativa* L. cv. SUSANA	4 dS m^−1^ (low) and 8 dS m^−1^ (high) NaCl; 60 d	CAT activity increased by 87 and 89% at low salinity, whereas at high salinity, it increased by 158 and 162% in both seasons. Elevation in POD, and PPO activities increased significantly.	[122]
*L. culinaris* Medik cv. BARI Masur-7	110 mM NaCl; 2 d	AsA content reduced by 70%, whereas an increase in GSH (305%) and GSSG (353%) contents was noticed. Reduction in CAT (71%) and APX (41%) activities, while an increase in DHAR (47%), GR (83%) and GPX (162%) activities.	[123]

**Table 3:** Effects of overexpressed antioxidant genes under salinity in different crops.

Transgenic Plants	Gene Source	Overexpressed Genes	Salt Stress and Duration	Regulatory Roles	References
*Nicotiana tabacum*	*A. thaliana*	*AtMDAR1*	300 mM NaCl; 6 d	Improved P*_n_*. Decreased H_2_O_2_ content.	[193]
*G. hirsutum*	*Agrobacterium tumefaciens*	*GhSOD1*, *GhCAT1*	200 mM NaCl; 2d	Upregulated SOD, CAT and APX activities. Increased oxidative stress tolerance.	[194]
*A. thaliana*	*Tamarix hispida*	*ThGSTZ1*	75, 100 and 125 mM NaCl; 12 d	Upregulated GST, GPX, SOD and POD activities. Reduced EL and MDA content.	[195]
*Arachis hypogaea*	*A. tumefaciens*	*SbpAPX*	50, 100, 150, 200 and 250 mM NaCl; 21 d	Increased chl content. Improved RWC. Increased plant biomass. Reduced EL.	[196]
*N. tabacum*	*A. hypogaea*	*AhCuZnSOD*	100 mM NaCl; 15 d	Increased RWC. Reduced MDA by 1.5-fold. Decreased H_2_O_2_ by 2-fold. Upregulated CAT, GR, APX and SOD activities.	[197]
*A. thaliana*	*Puccinellia tenuiflora*	*PutAPX*	125, 150 and 175 mM NaCl; 3 d	Improved total chl content. Increased RWC. Reduced MDA content. Upregulated APX activity.	[191]
*Ipomoea batatas*	*A. tumefaciens*	*CuZnSOD*, *APX*	100 mM NaCl; 14 d	Pro content increased by 2.7-fold. SOD and APX activities increased by 3.4- and 4.2-fold.	[198]
*S. tuberosum*	*Potentilla atrosanguinea*, *Rheum australe*	*PaSOD*, *RaAPX*	50, 100 and 150 mM NaCl; 15 d	Improved total chl content. Increased RWC. Upregulated SOD and APX activities.	[192]
*N. tabacum*	*S. lycopersicum*	*SlMDHAR*	200 mM NaCl; 5 d	Reduced MDA and H_2_O_2_. Reduced Na^+^ content.	[199]
*B*. *juncea*	*A. tumefaciens*	*AtApx1*	200 mM NaCl; 10 d	Increased chl and carotenoid content. Improved Pro content. Decreased MDA and H_2_O_2_ content. Increased APX and CAT activities.	[200]

## Data Availability

Not applicable.
